# Knowledge, Attitude, and Willingness Toward Kidney Donation Among Health Sciences Students at King Saud Bin Abdulaziz University

**DOI:** 10.3389/fpubh.2021.667582

**Published:** 2021-06-07

**Authors:** Raghad Sharaan, Sara Alsulami, Raneem Arab, Ghida Alzeair, Nadia Elamin, Basim Alsaywid, Miltiadis Lytras

**Affiliations:** ^1^King Saud Bin Abdulaziz University for Health Sciences, College of Medicine, Jeddah, Saudi Arabia; ^2^Urology Section, Department of Surgery, King Abdulaziz Medical City, Ministry of National Guard, Jeddah, Saudi Arabia; ^3^College of Medicine, King Saud Bin Abdulaziz University for Health Sciences, Ministry of National Guard, Jeddah, Saudi Arabia; ^4^Planning and Organizational Excellence Administration, Saudi Commission for Health Specialties, Riyadh, Saudi Arabia; ^5^Effat College of Engineering, Effat University, Jeddah, Saudi Arabia; ^6^Distinguished Scientists Program, Faculty of Computing and Information Technology, King Abdulaziz University, Jeddah, Saudi Arabia

**Keywords:** public health, Saudi Arabia, King Saud bin Abdulaziz University, willingness, kidney donation, Saudi Commission for Health Specialties, SCFHS

## Abstract

**Background:** End-stage renal disease, as one of the most serious and major health problems, does not have many treatment options available. One of the best treatment modalities used to cure this debilitating disease is kidney transplantation. However, with the continuous increase in number of patients diagnosed with it, there is not enough supply of the organ. The aim of our study is to assess knowledge about, attitude toward, and willingness to donate kidney among health science students at King Saud bin Abdulaziz University in comparison to the general population in Jeddah and to investigate the factors that play a role on their willingness.

**Methods:** This is an observational, analytical, cross-sectional study design conducted in 2019. Two target populations were included: King Saud bin Abdulaziz University for Health Sciences students and the general population in Jeddah. Data were collected via a self-administered, close-ended, structured, and previously validated questionnaire that contained 39 items divided into four sections. SPSS program version 22 was used in data analysis.

**Results:** Out of 685 surveyed participants, 179 (26.1%) were willing to donate their kidney, with students showing a higher rate of willingness (*N* = 101; 32.3%) than the general population (*N* = 78; 21%). However, only 46 (6.7%) out of the total population hold an organ donor card. In bivariate analysis, it was found that knowledge significantly associated with a higher rate of willingness among the student population than the general population, while positive beliefs were associated with increased willingness in the general population than students. Positive attitude appeared to play a role in higher willingness among the general population and student population.

**Conclusion:** There is a low perception of awareness regarding kidney donation in both populations of this study. The willingness rate of health science students at King Saud bin Abdulaziz University and the general population was low when compared with other studies conducted internationally.

## Introduction

The number of patients diagnosed with end-stage renal disease (ESRD) and documented on hemodialysis therapy in 2016 in Saudi Arabia was 16,315. This number has been significantly increasing over the years, and it is expected by the end of 2020 to go beyond 20,000. The age range of these patients is 26–65 years old (1), which is considered a relatively young age in contrast to the age groups of patients with other health conditions. This marks ESRD as one of the serious and major health problems that need to be highlighted. Those patients with ESRD could have different treatment options depending on their disease stage. Treatment options may include various types of dialysis; however, in ESRDs, the preferred treatment modality is kidney transplantation. Kidney transplantation is advised because it prolongs and improves the quality of life for patients who are enduring this debilitating condition. Also, it maintains a good level of cognitive function and enhances the patients' self-esteem due to the minimal disfiguring changes to the body (2). However, kidney transplantation is not always an available option due to the huge gap between the demand and the supply of the organ; therefore, out of the total number of patients on hemodialysis therapy, only 2708 are on the active waiting list (1, 3). Although the Saudi organ donation system has been improving in the recent years, implementation of new regulations could contribute to increase the rate of organ donation. For instance, a study done in Austria among the general population showed that several laws can be legislated to increase the rate of organ donation. One of these laws is to include all the population automatically in the organ donation system and give them the right to choose an opt-out option (a person has to inform formally that he/she does not want to donate his/her organ) (4).

Working on raising the number of kidney donors would make treatment with kidney transplant achievable for more patients, and it would lead to decreasing the discrepancy between the number of the patients who are on the waiting list and availability of kidney donors (5).

One of the important factors that play a major role in increasing the willingness to donate a kidney, which needs more focus and attention, is the attitudes and knowledge of health care students (6). The importance of focusing on this population lies behind the influence of their positive attitudes on the general population regarding the willingness toward kidney donation (7, 8). In addition, health care students are an important part of the community, and with their willingness to donate their kidneys, they set a good example for the public to follow. Some or many researchers found a correlation between increasing the donor pool and health care students' knowledge and education concerning kidney donation (8). However, other studies had claimed that other factors like religion and socioeconomic status play a more important role in the willingness to donate kidney than education level (2, 3, 7). In contrast, the result of a study done in Germany among medical students and physicians revealed a considerable association between the level of education and willingness to participate in organ donations. Moreover, the study showed that knowledge alone is poorly correlated with positive attitude toward organ donation (8). Furthermore, many studies showed a significant association between the knowledge, attitudes, and increase of the willingness to donate a kidney (9). However, there is not sufficient research done in Saudi Arabia, particularly in Jeddah, that looks into this topic.

The recent literature is also providing interesting insights for the justification of our research and its focus. In the table below, we provide a synthesis of the recent review on organ donation research. We should say from the beginning that we do plan to deliver one more sophisticated research soon that will focus on cultural and religious issues associated with organ donation.

**Literature review:** 

**References**	**Title of article**	**Key contribution**	**Impact on our research model**
Ehlers et al. (10)	Altruistic Non-directed Kidney Donation: Attitudes, Characteristics and Ethical Implications.	**Focus:** The mental health of altruistic non-directed kidney donors. •The general attitude toward the practice of this form of donation. •The willingness of the public to be altruistic non-directed kidney donors.	•Willingness •Added Value of Organ Donation
Ríos et al. (11)	Differences in Attitudes Toward Living Kidney Donation Among Dominican Immigrants Living in Spain and the United States.	**Focus**: To determine attitudes toward living organ donation among Dominicans residing in Florida (USA) and Spain. **Participants**: 123 Dominicans	•Understanding of attitudes
Zhang et al. (12)	The Family Attitudes of Patients With End-Stage Renal Disease Toward Living Kidney Donation in China.	**Focus:** To analyze the family attitudes of patients with ESRD toward living kidney donation in China. **Participants:** Hemodialysis and nephrology patients of 5 Chinese 5 third-level hospitals.	•Understanding of attitudes
Loughery et al. (13)	Organ Donation Attitudes Among Individuals With Stage 5 Chronic Kidney Disease.	**Focus:** To explore beliefs of individuals with stage 5 CKD about their ability to donate and test the validity of an organ donation scale. **Participants:** 554 patients at 12 dialysis units in southeast Michigan	•Codification of beliefs and knowledge toward organ donation
Ríos et al. (14)	Attitudes of Latin American Immigrants Resident in Florida (United States) Toward Related Living Kidney Donation.	**Focus:** •To analyze the attitude toward living kidney donation •To identify the psychosocial variables affecting patients' attitude. •**Participants:** Latin American residents in the state of Florida	•Analysis of living kidney donation
Bieniasz et al. (16)	Psychological Aspects of Living Kidney Donation in Poland: Experience of One Center.	**Focus:** To evaluate the psychological aspects of living kidney donation in Poland. •**Participants:** 40 Polish donors after nephrectomy.	•Analysis of psychological impact of living kidney donation
von Zur-Mühlen et al. (17)	Few Gender Differences in Attitudes and Experiences after Live Kidney Donation, with Minor Changes Over Time.	**Focus:** To study gender differences and differences over time compared with •demographics •donors motives •experiences of live kidney donation. •**Participants:** 387 consecutive live kidney donors, representing all of the donors of one donation center between 1974 and 2008.	•Demographics and Donors motivation analysis
Ríos et al. (18)	Living Kidney Donation Questionnaire (PCID-DVR-Ríos): Validation and Psychometric Characteristics in a Spanish-Speaking Population.	**Focus:** To analyze the psychometric characteristics of the attitudes questionnaire about living renal donation. (PCID-DVR-Ríos). •**Participants:** Over 18 years old native and resident Spanish.	•Analysis of psychometric characteristics of attitude toward organ donation
Agwu et al. (15)	Awareness and attitude to deceased kidney donation among health-care workers in Sokoto.	**Focus:** To assess the awareness and attitude to deceased kidney donation. •**Participants:** 470 health-care workers in Sokoto, Nigeria.	•Awareness and attitude •Decreased rate of kidney donation
Ríos et al. (19)	Factors That Condition the Attitude Toward Living Related Kidney Donation Among Santiago of Cuba‘s Population.	**Focus:** •To analyze the attitude toward living kidney donation. •To determine the sociopersonal factors. **Participant:** Over 15 years old residing in Santiago de Cuba.	•Socioperonal factors (religion)
Lafranca et al. (20)	Attitudes among Transplant Professionals regarding Shifting Paradigms Ineligibility Criteria for Live Kidney Donation.	**Focus:** •To reveal the geographical differences in policies regarding the acceptance of donors. •To get an insight into both center policies as well as personal beliefs of transplant professionals. •**Participants:** 1,128 ESOT-members.	•Geographical **differences** •Policies formulation
Topbaş and Taştan (21)	Does Having a Relative in Dialysis Therapy Affect Attitudes Toward Kidney Donating?	**Focus:** To compare the view points and the attitudes of individuals who have relatives undergoing dialysis toward kidney donation treatment with those who do not. •**Participants:** Two groups of 204 individuals in Turkey.	•Family issues and percpectives
Trachtman et al. (22)	Physician attitudes toward living kidney donation, Progress in Transplantation.	**Focus:** To ascertain Physicians' attitudes toward living donation. **Participants:** Surveyed 104 physicians, pediatric, and internal medicine nephrologists.	•Attitudes of physicians
de Jong et al. (23)	Results of the European Effect of Differing Kidney Disease Treatment Modalities and Organ Donation and Transplantation Practices on Health Expenditure and Patient Outcomes nephrologist survey on factors influencing treatment modality choice for end-stage kidney disease.	**Focus:** To survey European nephrologists and kidney transplant surgeons about factors choice to provide treatments for ESKD. **Participants:** 681 professionals from 33 European countries	•Kidney Donation and transplantation

The key summary of perspectives and focus on recent literature on organ donation is provided below. The list is not exhaustive but provides a rich picture of the research approaches and objectives:

- The willingness of public for kidney donation (10)
- The positive impact on mental health of kidney donors (10)
- The attitudes and perceptions of residents toward living organ donation (11)
- To understand family attitudes of patients with ESRD toward living kidney donation (12)
- To explore beliefs of individuals about their ability to donate and test the validity of an organ donation scale (13–15)
- To identify the psychosocial variables affecting patients' attitude (14)
- To evaluate the psychological aspects of living kidney donation in Poland (16)
- To study gender differences and differences over time compared with demographics, donors' motives, and experiences of live kidney donation (17)
- To analyze the psychometric characteristics of the attitudes questionnaire about living renal donation (18)
- To assess the awareness and attitude to deceased kidney donation (15)
- To determine the socio-personal factors (19)
- To reveal the geographical differences in policies regarding the acceptance of donors (20)
- To compare the viewpoints and the attitudes of individuals who have relatives undergoing dialysis toward kidney donation treatment with those who do not (21)
- To ascertain physicians' attitudes toward living donation (22)
- To survey European nephrologists and kidney transplant surgeons about factors and choice to provide treatments for ESKD (23).

The aim of this study is to assess awareness, attitude, and willingness among health science students at King Saud bin Abdulaziz University in Jeddah toward kidney donation and to investigate the factors that contribute to kidney donation willingness or opposition. Another goal that will be achieved is comparing the responses of the students with the general population.

## Methods

This study was approved by the Institutional Review Board of King Abdullah International Medical Research Center (KAIMRC) with the registered number (H-01-R-005) and informed written consent was taken from all participants. This is an observational analytical cross-sectional study design where invited participants were grouped into two main groups; group 1 consists of students of health sciences and group two consists of the general population. All invited participants filled up a self-administered, close-ended, structured, previously validated questionnaire to identify the willingness toward kidney donation and to compare the students of King Saud bin Abdulaziz University for Health Sciences (KSAU-HS) with the general population. The inclusion criteria for the study included adults, which were defined as more than 18 years of age, lives in Jeddah, and both genders. The sample was grouped according to the settings into health science students at KSAU-HS and the general population who live in the same city. The students included were from all health science colleges including College of Medicine, Nursing, and Applied Medical Science at KSAU-HS, while data for the targeted group two who lives in Jeddah were collected from people who went to major malls distributed across the city. The sample size for each group was calculated to be around 314 participants, and the sampling technique design was convenient non-probability sampling technique.

The questionnaire tool utilized in this study is composed of four domains. The first domain was to determine the basic characteristics of the participants. The second domain was to assess the level of knowledge, the third domain was to assess the attitude, and the fourth domain was to assess the beliefs regarding kidney donation among the participants. It consisted of 34 items: seven items assessing demographics, nine items measuring knowledge, seven items measuring attitude, and 11 measuring beliefs. The items regarding knowledge used multiple-choice questions. The items regarding attitude and beliefs used answers on a five-point Likert scale, where possible responses range from “strongly agree” to “strongly disagree.” For each knowledge question, a correct response was assigned a score of “1,” and an incorrect response was assigned a score of “0.” The total knowledge score was obtained by adding the scores for all knowledge responses. Then, an “overall knowledge percent score” was calculated by multiplying the total knowledge score for each participant by 100 and dividing the product by nine. Scores from 0 to 2 were assigned for attitude and beliefs statements. Responses ranged from strongly agree to strongly disagree. A “strongly agree” or “agree” response was assigned a score of “2” while a “strongly disagree” or “disagree” response was assigned a score of “0.” A “neither agree nor disagree” (neutral) response was assigned a score of “1”. A total attitude or beliefs score was obtained by adding the scores for all attitude or beliefs responses. Then, an “overall attitude percent score” or “overall beliefs percent score” was calculated by multiplying the total attitude score for each participant by 100 and dividing the product by 7 or 11, respectively.

The data were analyzed using SPSS version 22 program. Simple descriptive analysis was reported as frequency and percentage for qualitative data, while mean and standard deviation were used for normally distributed quantitative data and median and interquartile range were used for skewed data. For inferential statistic or bivariate analysis, chi-square test was reported for qualitative data, and for the quantitative data, independent sample *t*-test and Mann–Whitney test were used. *P* < 0.05 was considered significant.

## Results

The study population was divided into two groups. The first group consists of health science students at KSAU (*N* = 313; 45.7%), while the second group consists of the general population (*N* = 372; 54.3%).

Studying the baseline characteristics of the sample population, the median age was 22 years (IQR = 9), the dominant gender was female with a total of 502 participants (73.3%), and the marital status of the majority of the respondents was single 474 (69.2%). Also, majority of the general population were non-governmental employees 96 (25.8%) followed by students 90 (24.2%). The income of most of the participants was reported to be < SR10,000/months (*N* = 226; 33%) or between SR10,000 and 20,000/months (*N* = 208; 30.4%). When asked about education level, the preponderance of the population answered that they have completed secondary education (*N* = 411; 60%). The separated characteristics of the two groups are shown in Table 1.

**Table 1:** Demographic data.

**Participant characteristic**	**Students *N* (%)**	**General population *N* (%)**	***P*-value**
Age (median, IQR^*^)	21 years (2)	31.10 years (14)	
Gender			0.001
Female	201 (64.2)	301 (80.9)	
Male	112 (35.8)	71(19.1)	
Job sector			0.001
Student	313 (100)	90 (24.2)	
Housewife		74 (19.9)	
Government employee		72 (19.4)	
Non-government employee		96 (25.8)	
Others		40 (10.8)	
Income			0.002
< SR 10,000/months	102 (32.6)	124 (33.3)	
SR 10,000–20,000/months	70 (22.4)	138 (37.1)	
SR 20,100–SR 30,000/months	43 (13.7)	36 (9.7)	
SR 30,000 and above	42 (13.4)	27 (7.3)	
Refused not to elect	56 (17.9)	47 (12.6)	
Collage			0.001
College of medicine	150 (47.9)		
College of nursing	36 (11.5)		
College of applied medical science	126 (40.3)		
Marital status			0.001
Single	300 (95.8)	174 (46.8)	
Married	11 (3.5)	162 (43.5)	
Others	2 (0.6)	36 (9.7)	
Level of education			0.001
Primary		2 (0.5)	
Intermediate		13 (3.5)	
Secondary (till class 12 or equivalent)	276 (88.1)	136 (36.6)	
Graduation (bachelor and diploma)	34 (10.9)	197 (52.9)	
Post-graduation (Master and doctoral)	3 (1)	24 (6.4)	

* *Interquartile range*.

### General Inquiry About Organ Donation

The term organ donation was known to 661 of the sample population (96.5%), and the Internet represents the most common method through which they have heard about it from 313 (45.7%). Based on the data of our survey, 25.2% of our respondents have attended organ donation promotion campaigns in Saudi Arabia. It was shown that only 179 (26.1%) were willing to register as a kidney donor in Saudi Arabia, and this number represents the minority of the study participants. Moreover, only 46 (6.7%) reported being already registered as an organ donor. The general inquiry of the two different groups is shown in Table 2.

**Table 2:** General inquiry about organ donation.

**General inquiry**	**Students *N* (%)**	**General population *N* (%)**	***P*-value**
1. Have you ever heard of the term “Organ donation”?			0.667
Yes	301 (96.2)	360 (96.8)	
No	12 (3.8)	12 (3.2)	
2. How did you hear about organ donation			
Word of mouth	96 (30.7)	97 (26.1)	0.183
Newspaper	26 (8.3)	55 (14.8)	0.009
Television	81 (25.9)	119 (32)	0.092
Radio	23 (7.3)	21 (5.6)	0.365
Internet	155 (49.5)	158 (42.5)	0.077
Social event and social get together	89 (28.4)	89 (23.9)	0.568
Other	55 (17.6)	41 (11)	0.796
3. Have you ever attended organ donation promotion campaigns in Saudi Arabia			0.001
Yes	64 (20.4)	18 (4.8)	
No	249 (79.6)	354 (95.2)	
4. Are you willing to register as a kidney donor in Saudi Arabia			0.001
Yes	101 (32.3)	78 (21)	
No	212 (67.7)	294 (79)	
5. Are you registered as an organ donor			0.001
Registered	33 (10.5)	13 (3.5)	
Not registered	280 (89.5)	359 (96.5)	

### Knowledge About Kidney Donation

**Figure 1:**
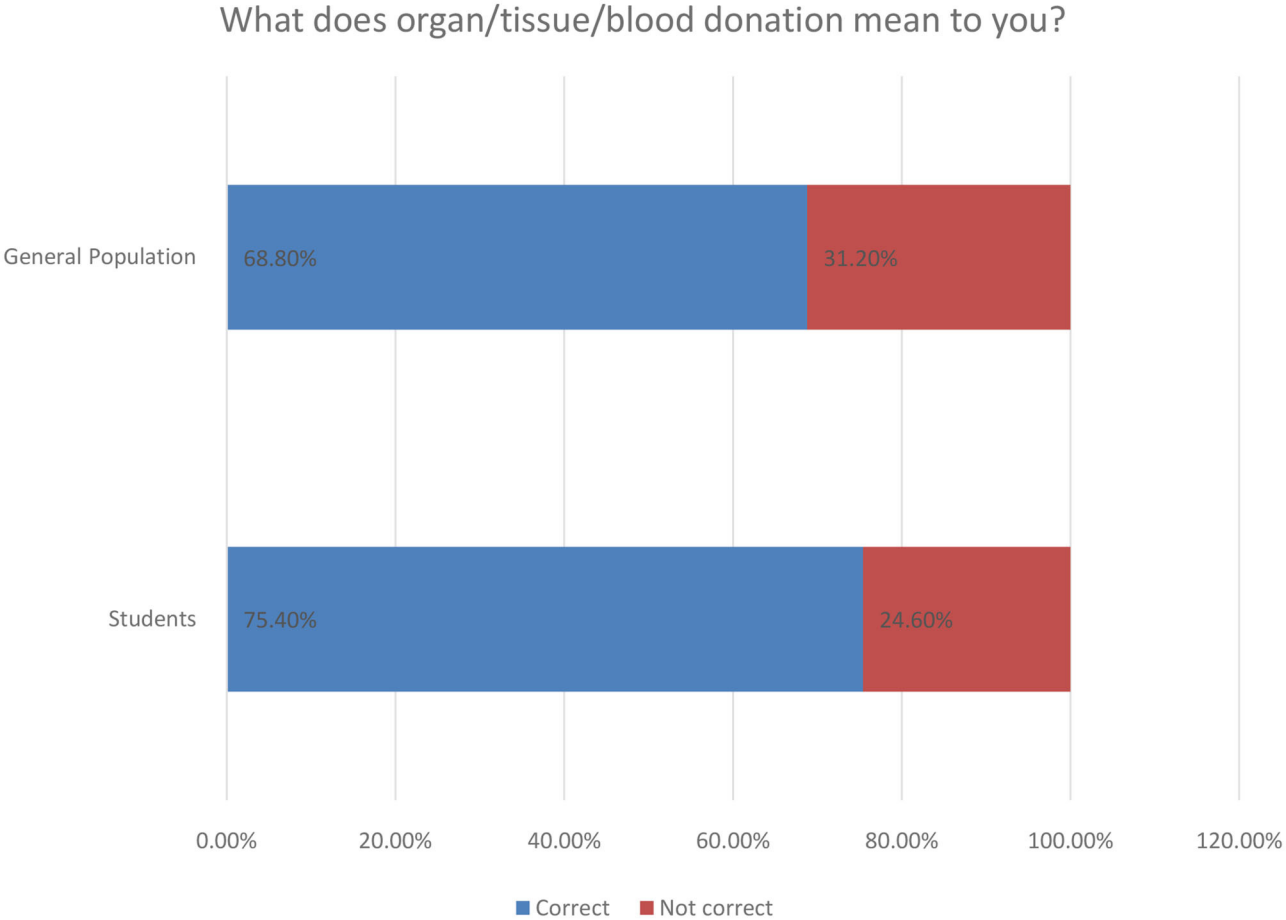
Attitude toward organ/tissue/blood donation.

The knowledge about kidney donation among the participants was assessed through multiple questions, which consisted of nine close-ended questions, and they were scored by the method that was mentioned previously in the *Methods* section. There was a significant difference between the score means of the two populations with *P* < 0.0001, which is shown in Table 3. The mean score of the students was 8.330 ± 1.861 in comparison to 7.758 ± 7.0555, which represent the mean score of the general population. The questions that were used to assess the knowledge are the following: The concept of organ/tissue/blood donation was understood correctly by 493 (72%). About 349 (51%) of the participants have not ever heard about Saudi organ donation registry. Almost half of the participants do not know that the minimum legal age to register for kidney donation is 18 years (*N* = 351; 51.2%). In Figure 1 below, we provide an overview of the basic aspects of knowledge of respondents related to organ/tissue and blood donation.

**Table 3:** Knowledge about kidney donation.

**Knowledge about kidney donation**	**Students *N* (%)**	**General population *N* (%)**	***P*-value**
1. What does organ/tissue/blood donation mean to you?			0.057
Correct	236 (75.4)	256 (68.8)	
Not correct	77 (24.6)	116 (31.2)	
2. There is a donor registry in Saudi Arabia where people register during their life to donate organs after death. Have you heard about it?			0.001
Yes	191 (61)	145 (39)	
No	122 (39)	227 (61)	
3. At what age can an individual register for kidney donation?			0.012
Correct	169 (54)	165 (44.4)	
Not correct	144 (46)	207 (55.6)	
4. Death could mean:			0.001
Correct	104 (33.2)	59 (15.9)	
Not correct	209 (66.8)	313 (84.1)	
5. Does your religion allow organ donation?			0.116
Yes	234 (74.8)	261 (70.2)	
No	15 (4.8)	14 (3.8)	
Don't know	64 (20.4)	97 (26.1)	
6. Do you know anyone who has donated a kidney?			0.001
Yes	74 (23.6)	150 (40.3)	
No	239 (76.4)	222 (59.7)	
7. Do you know that you can donate one of your two kidneys during your life, to another person?			0.230
Yes	275 (87.9)	315 (84.7)	
No	38 (12.1)	57 (15.3)	
8. Do you know that donating a kidney is safe?			0.018
Yes	184 (58.8)	185 (49.7)	
No	129 (41.2)	187 (50.3)	
**Saudi organ donation law and policy**			
1. Prohibits any buying or selling of organs:			0.001
Yes	250 (79.9)	245 (65.9)	
No	63 (20.1)	127 (34.1)	
2. Provides access to transplant facility for all nationalities equally:			0.079
Yes	165 (52.7)	221 (59.4)	
No	148 (47.3)	151 (40.6)	
3. Gives donated organs from deceased donors to the first person on the waiting list regardless of nationality:			0.717
Yes	202 (64.5)	245 (65.9)	
No	111 (35.5)	127 (34.1)	
4. Puts no pressure on the deceases donor family or living donor to donate:			0.917
Yes	235 (75.1)	278 (74.7)	
No	78 (24.9)	94 (25.3)	
5. All live donors in Saudi Arabia are provided with health insurance for life:			0.938
Yes	101 (32.3)	119 (32)	
No	212 (67.7)	253 (68)	
6. All families of the deceased in Saudi Arabia will receive social support if they need it:			0.001
Yes	139 (44.4)	215 (57.8)	
No	174 (55.6)	157 (42.2)	

**Figure 2:**
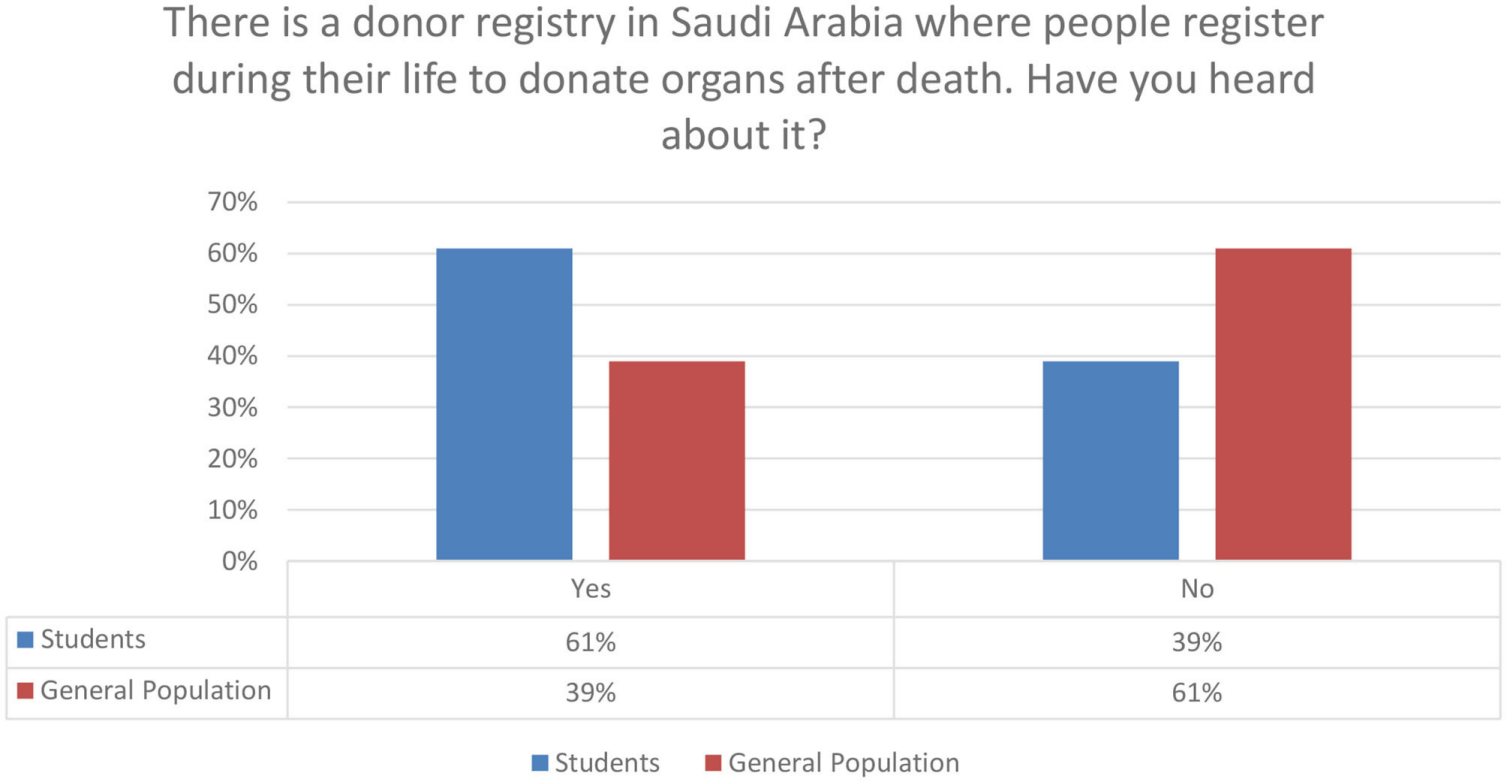
Knowledge about donor registry in Saudi Arabia.

In Figure 2 below, we also summarize the awareness of respondents for the donor registry in Saudi Arabia. It is evident that students in comparison to the general population have a much higher awareness.

**Figure 3:**
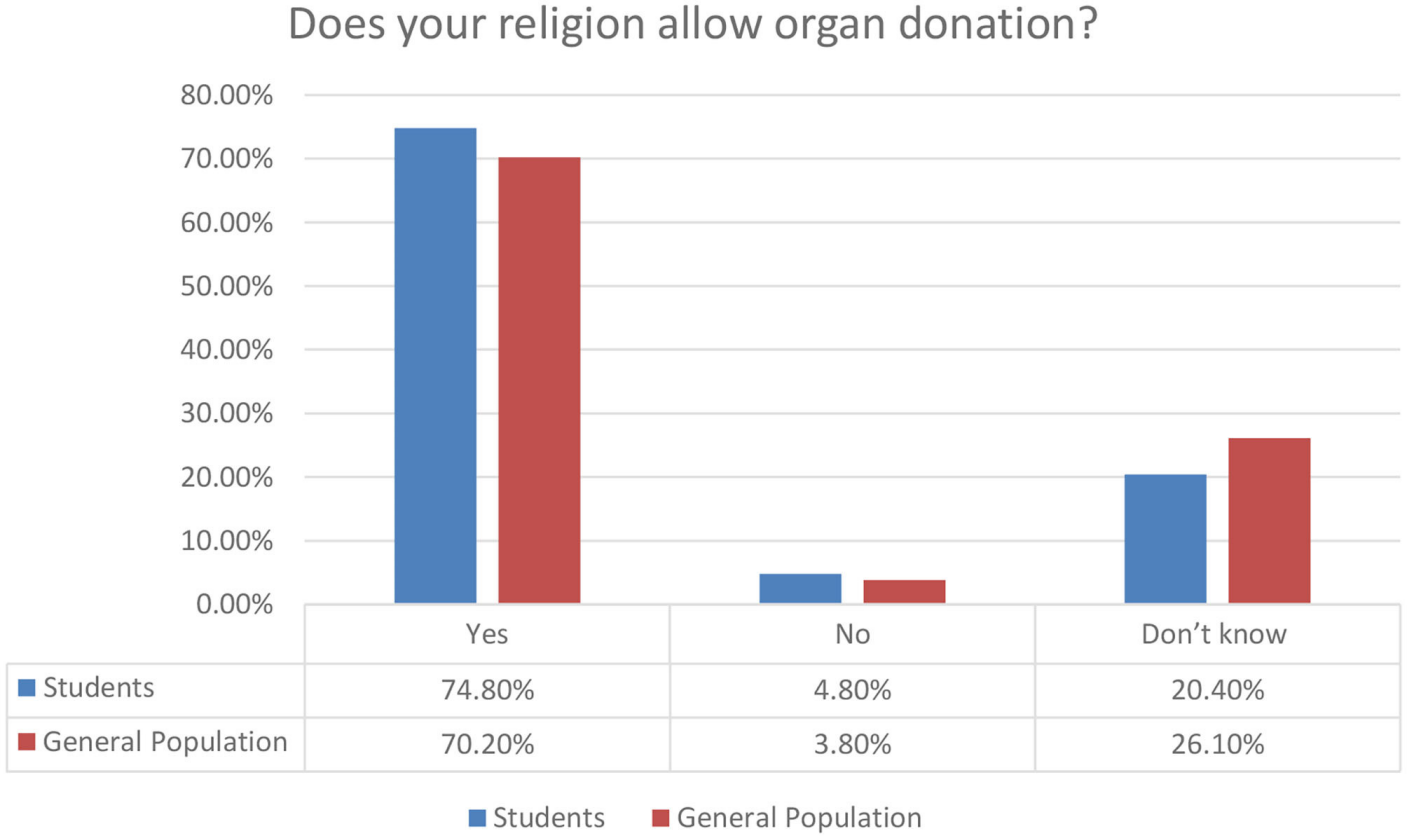
Religion impact on organ donation perceptions.

To understand better the attitude of respondents and the impact of religion to its formulation, we provide Figure 3. It is evident that most respondents confirm broadly that religion does not formulate barriers for organ donation. Almost one fifth of the students and one fourth of the general population have no knowledge about the connection between religion and organ donation. This study does not focus on the interpretation of this aspect, but we do plan a future research on this matter.

**Figure 4:**
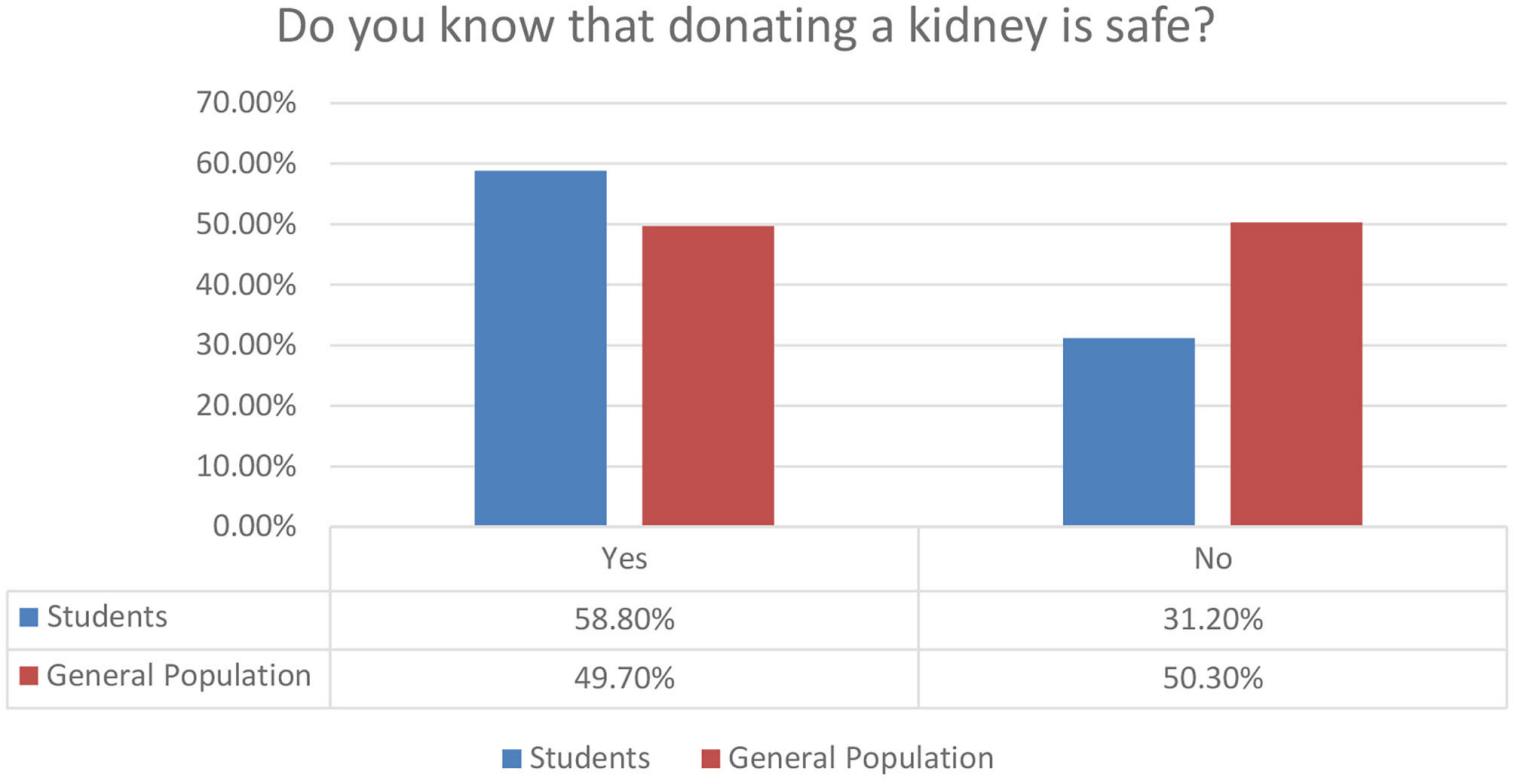
Perception on safety for kidney donation.

The participants of our research also communicate their concerns about the safety of kidney donation. In Figure 4, it is depicted that almost half of the general population respondents are worried about the safety aspect. An implication for future campaigns is that the public should be informed about the safety and the efficiency of modern methods that will be applied to individuals who donate organs.

After asking about death definition, 522 (76.2%) gave an incorrect answer. More than 495 (72.3%) of subjects believe that their religion does not prohibit organ donation. Most of our population were not acquainted with someone who donated a kidney (*N* = 461; 67.3%). When the population was asked if they know that donating one of the kidneys is possible, 590 (86.1%) answered “yes,” and 369 (53.9%) are aware that the transplantation process is safe. After asking the participants about the Saudi organ donation laws and policies, 494 (72.1%) know that buying or selling of organs is prohibited in Saudi Arabia, 385 (56.2%) know that it provides access to transplant facility for all nationalities equally, 447 (65.3%) know that it gives the donated organ to the first patient on the waiting list regardless of the nationality, 513 (74.9%) know that it puts no pressure on the donor family to donate, 465 (67.9%) know that it does not provide all live donors insurance for life, and 331 (48.3%) know that all families of the deceased in Saudi Arabia will not receive social support if they need it.

In the next section, we elaborate further on the key findings of our survey with an emphasis on the attitude toward kidney donation.

### Attitude Toward Kidney Donation

After asking seven questions to assess the attitude, it was surprisingly found that both the students and the general population scored similarly with comparable means represented by *P* = 0.393. The general population attitude score mean was 4.978 ± 1.437, whereas the students' score mean was 4.853 ± 1.696. A large percentage of our study population showed positive attitudes toward kidney donation, 548 (80%) believe that kidney donation should be promoted, 634 (92.6%) believe that donating a kidney could save someone's life, and 410 (59.9%) agree that Saudi and non-Saudi residents should automatically be included in the organ donation registry. Moreover, a large number would be more willing to register as a kidney donor if their families show no objection (*N* = 451; 65.8%), if more information about kidney transplantation were available (*N* = 457; 66.7%), if the viewpoint of their religion was clear (*N* = 464; 67.7%), and if they knew where they could register (*N* = 423; 61.8%).

**Figure 5:**
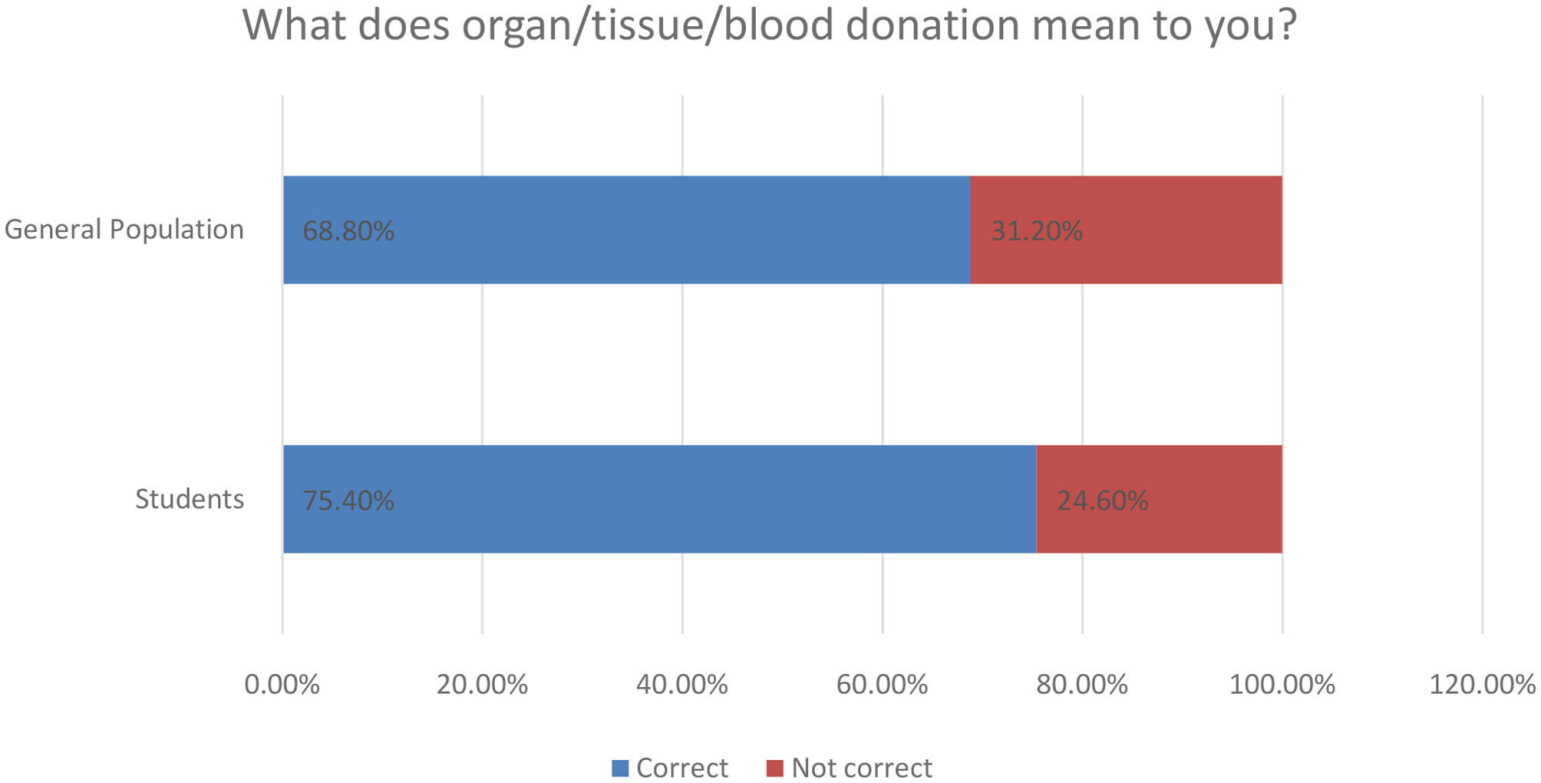
Personal interpretation of kidney donation for respondents.

In Figure 5 below we provide the general interpretation of respondents concerning organ donation action. As an overall comment, almost 75% of students find it correct and the same perception is rated at 68.8% for the general population cluster of our research.

**Figure 6:**
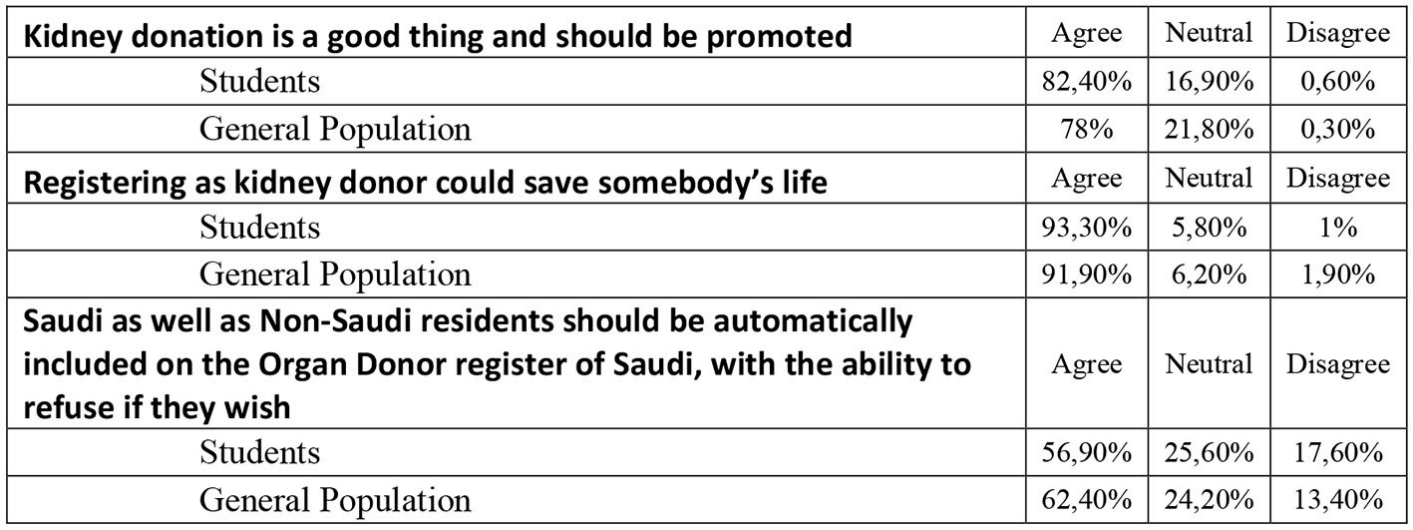
Value perception of kidney donation.

In Figure 6, we provide some key results related to the value perception of respondents associated with kidney donation. Most respondents in both clusters of our research (students and general population) view organ donation as a good thing that can save somebody's life.

It is also very encouraging that the majority of respondents agree to the automatic inclusion of residents in the Organ Donor Registry. In the next section, we provide additional insights into the beliefs of respondents.

### Beliefs Toward Kidney Donation

In the section of the questionnaire that assesses the beliefs that favor and encourage kidney donation, it was shown that the general population has a higher mean score (14.833 ± 2.519, in comparison to students, 13.403 ± 2.937; *P* < 0.002). In spite of that, three out of 11 questions that we use to study the beliefs had no significant difference between the two groups. A considerable number of the respondents believe that kidney donation is going to impact their life after death in a good way (*N* = 540; 78.8%) and that it will be rewarded by God (*N* = 604; 88.2%). However, 259 (43.1%) were concerned that kidney donation might leave them weak and disabled, and 360 (52.6%) were concerned about their families' emotion. A total of 255 (37.2%) respondents think that they are healthy enough to donate and that their age is appropriate for the process (*N* = 319; 46.6%). A larger number of participants were neutral about the idea that the kidney retrieval process may cause body disfigurement (*N* = 270; 39.4%), about not finding many opportunities to register (*N* = 290; 42.3%), about the idea that the kidney retrieval process is time-consuming (*N* = 351; 51.2%), about not being able to get answers for their questions regarding the kidney donation process (*N* = 312; 45.5%), and about the fact that the whole process is discouraging (*N* = 322; 47%).

**Figure 7:**
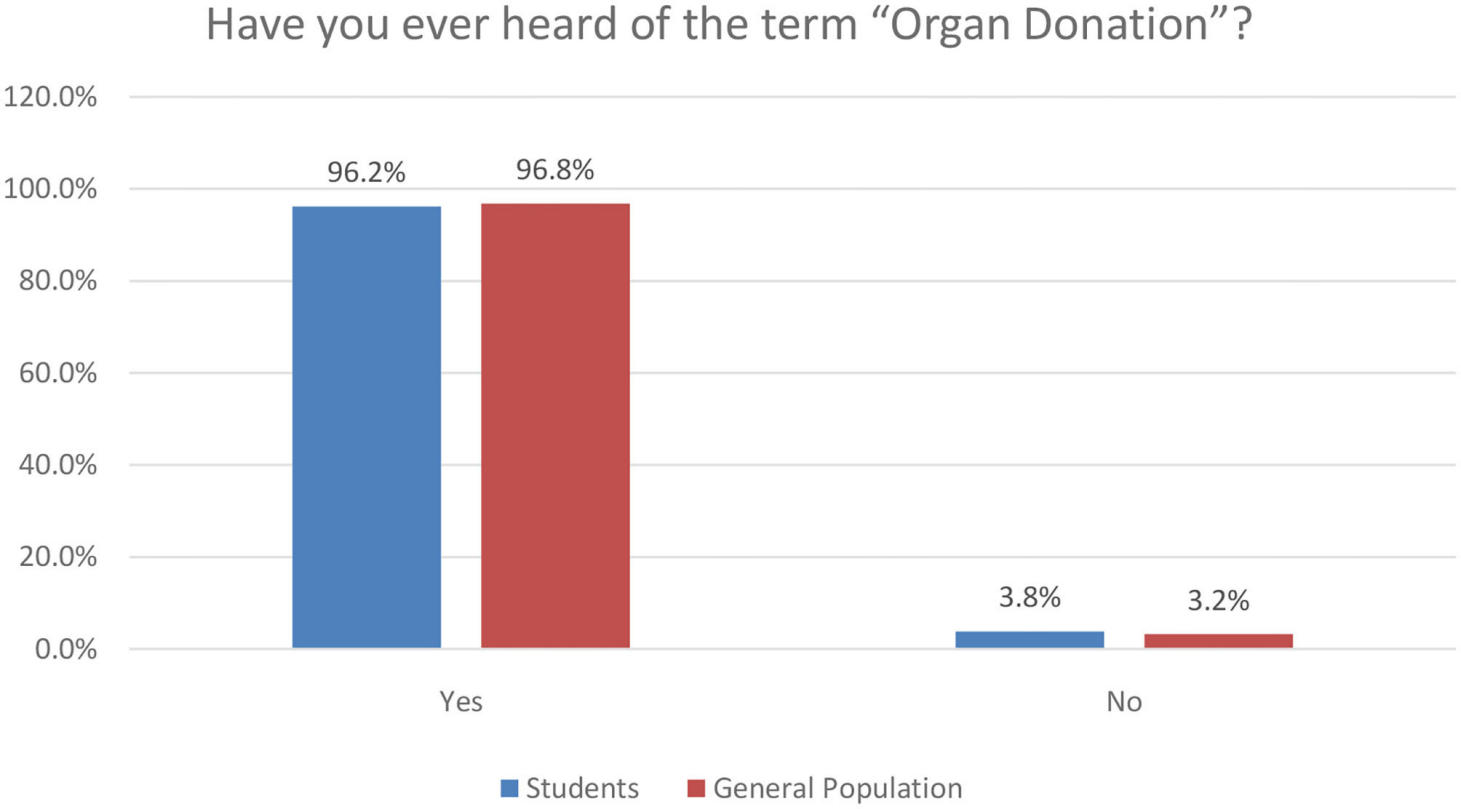
Awareness on organ donation.

**Figure 8:**
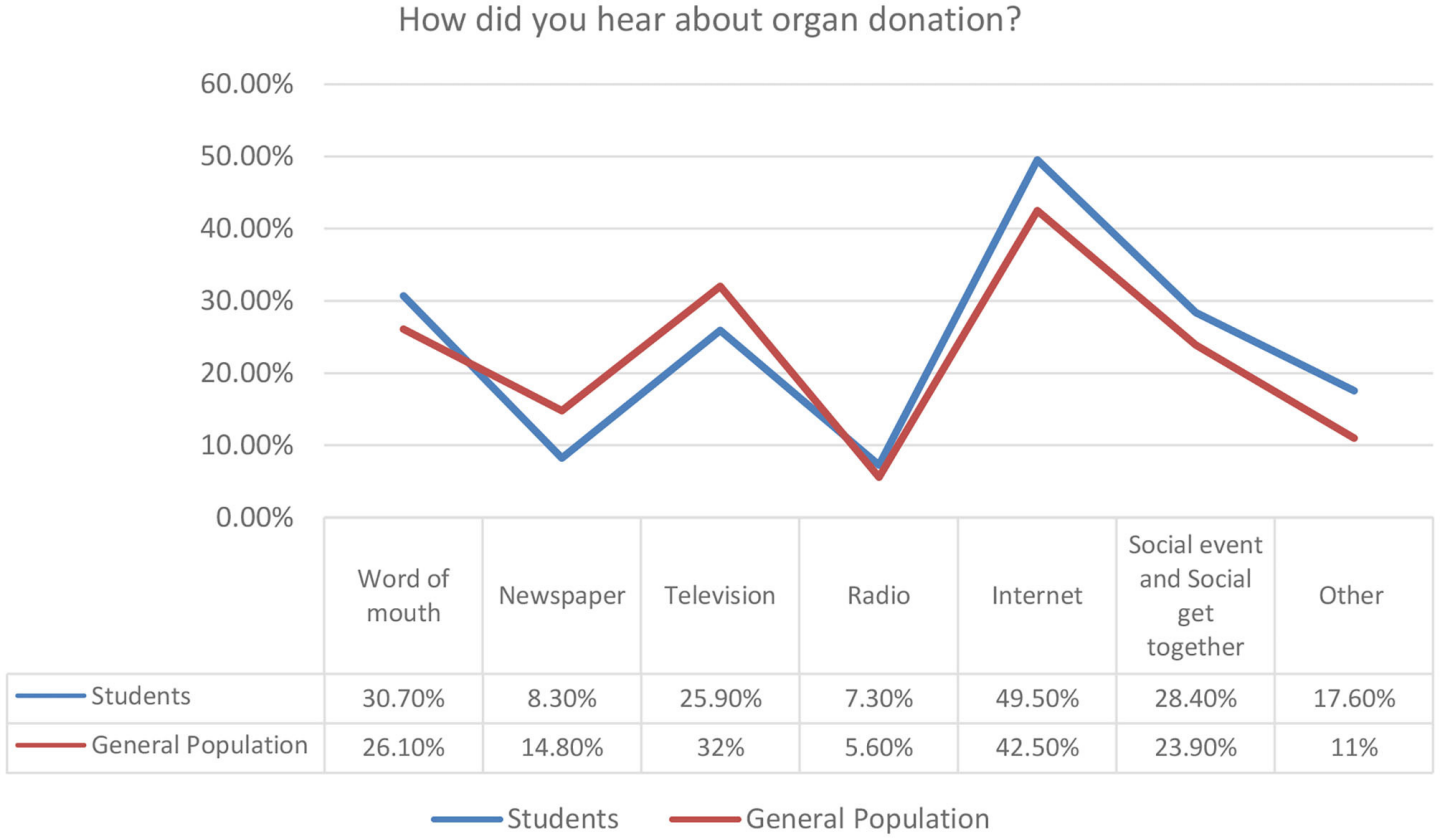
Sources of awareness for organ donation campaign.

**Figure 9:**
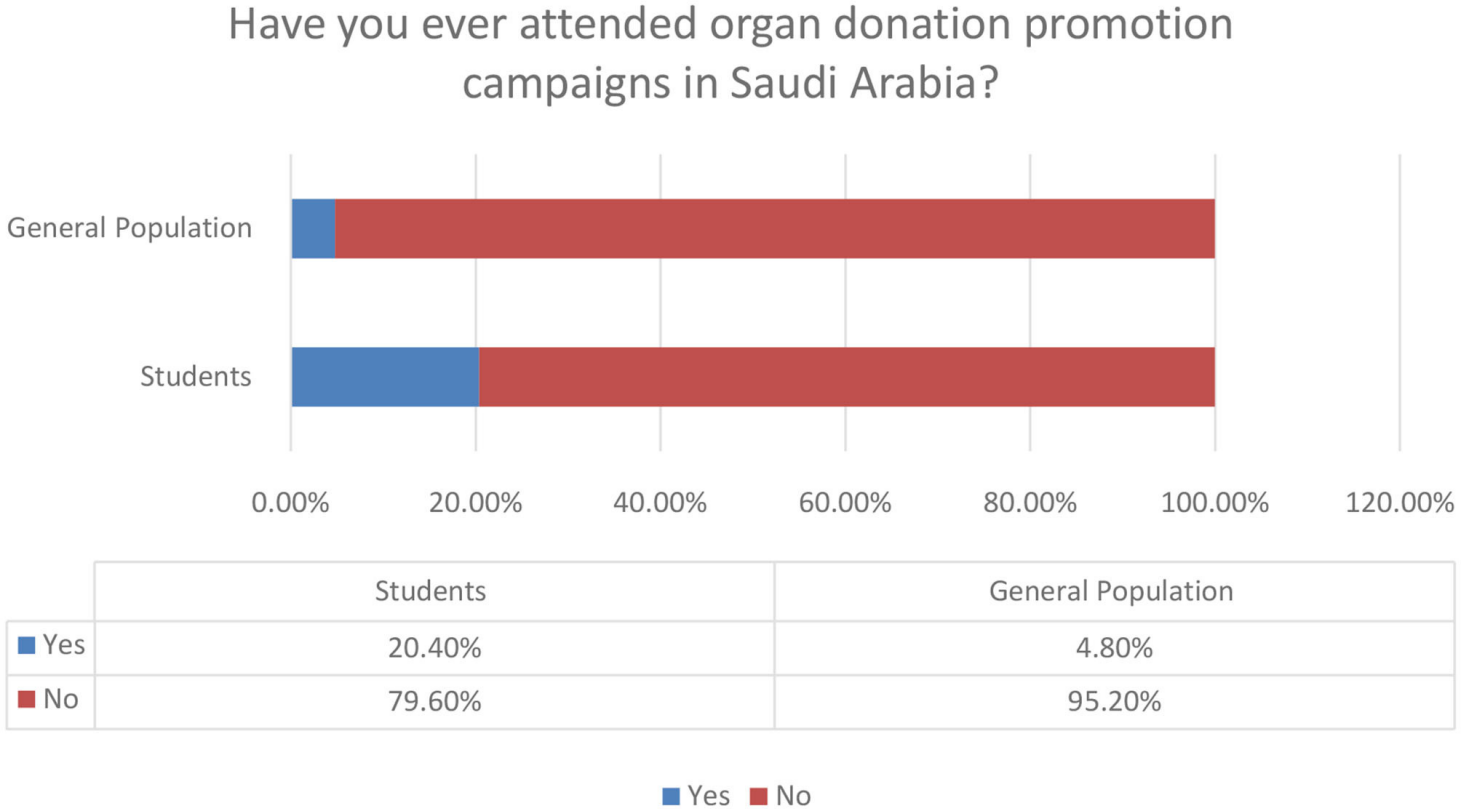
Personal attendance in organ donation promotion campaigns.

**Figure 10:**
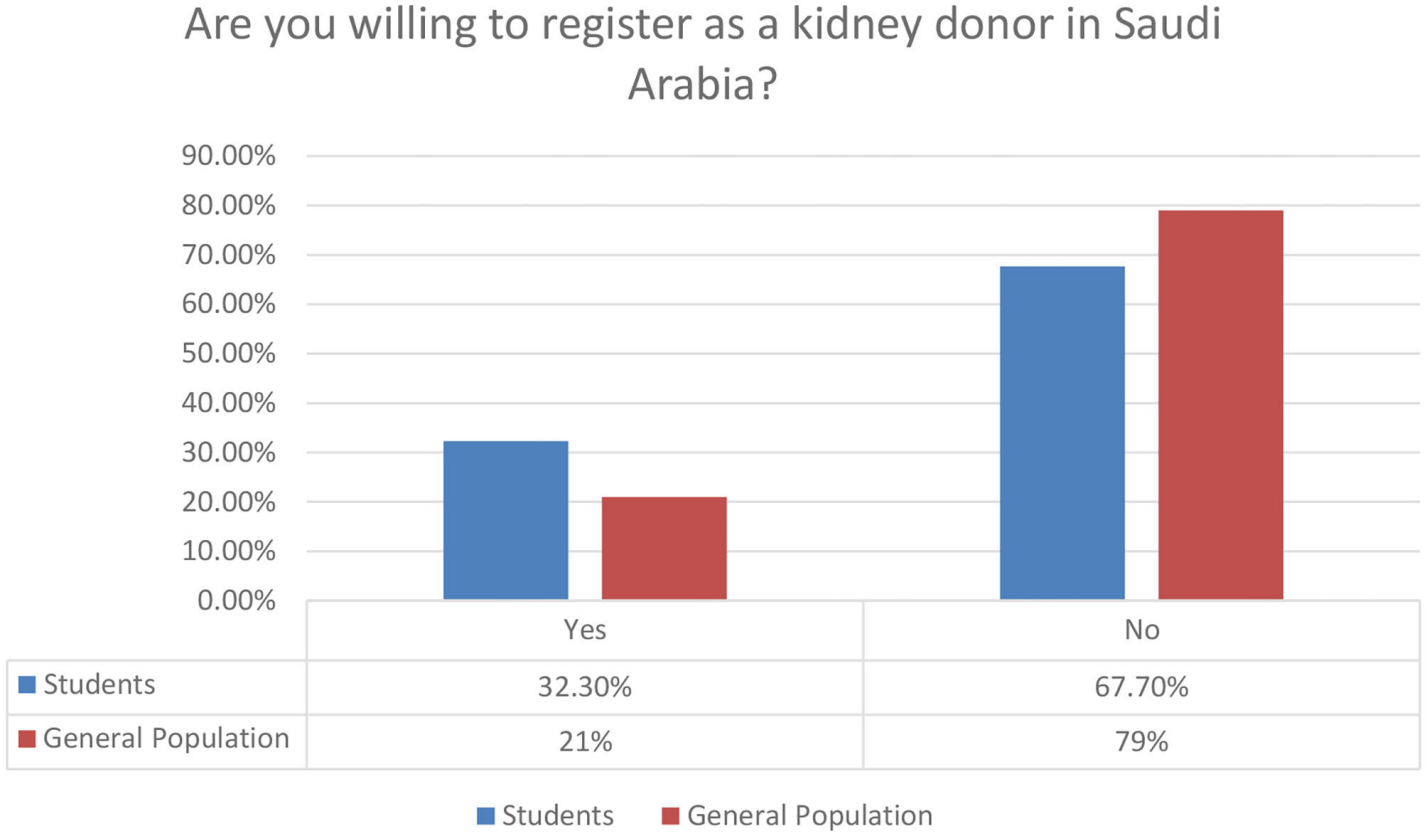
Personal willingness to register as a kidney donor.

**Figure 11:**
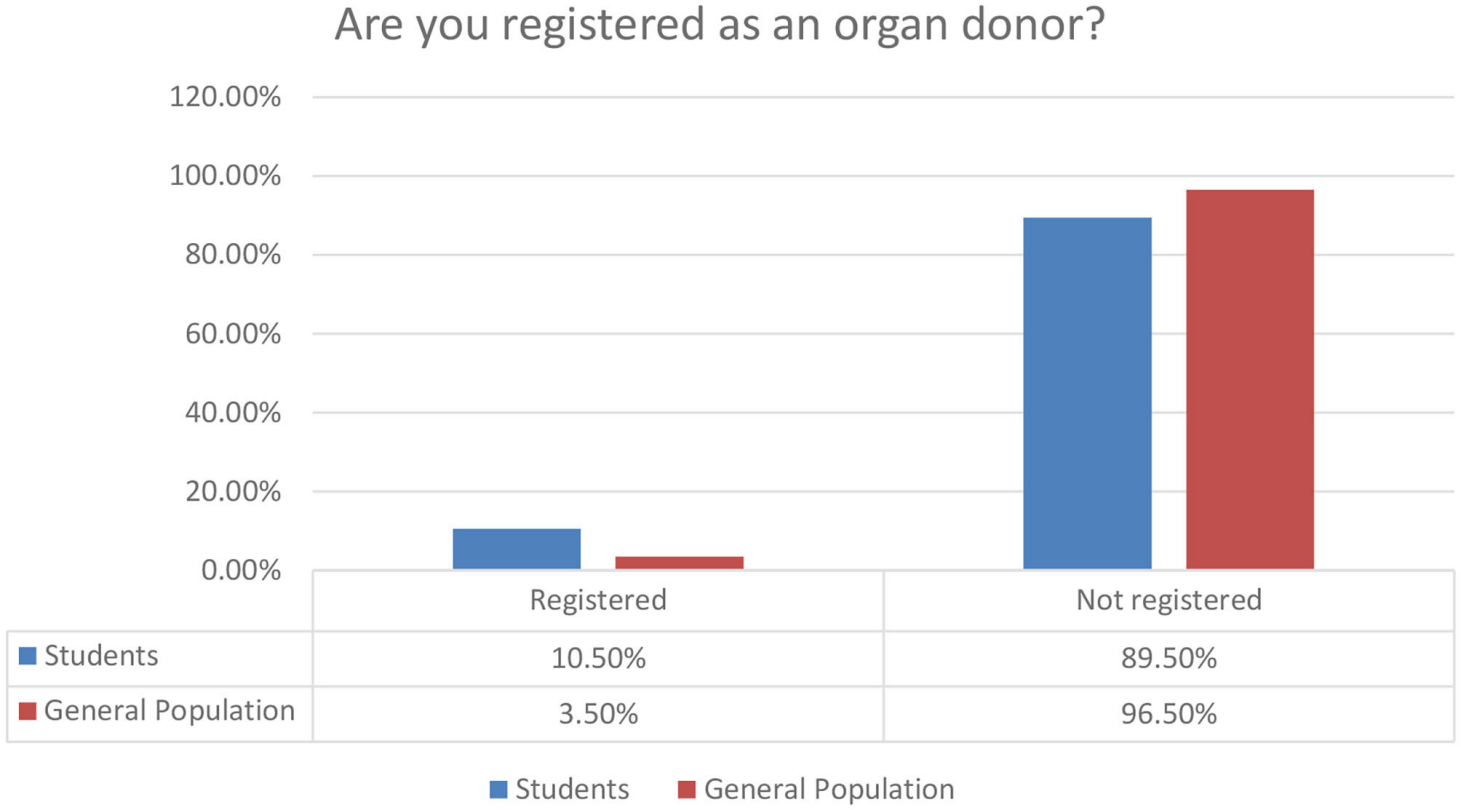
Organ donor registry status.

In Figure 7–11 below, we summarize some additional key findings of our research. The overall positive finding is that currently students and the general population have a significant awareness for organ donation. This is positive and it is important to understand how this general awareness is transformed to official registries and increased willingness for organ donation.

In Figure 8, we summarize also the key sources for the increased awareness on organ donation. Internet is confirmed as an effective means for campaigns, and word of mouth and television also appear to be high sources of awareness. Some minor variations between student and general population clusters are depicted, but the overall trend is the same. From this point of view, future campaigns should take into consideration these findings.

The personal experience of respondents in attending organ donation promotional campaigns is confirmed at extremely low rates for the general population and higher for students that participated in our research. Only 4.8% for the general population and 20.40% for students are the confirmed rates. From this point of view, it seems that there is a lot of space for improvement in the rates of personal involvement in organ donation activities in Saudi Arabia.

In Figure 10, we provide a graphical overview of the personal willingness of our participants to register as kidney donors. The relevant percentages of 32.3 and 21% for student and general population clusters, respectively, are encouraging. We do believe though that additional promotional activities for organ donation can result to much higher rates soon.

One more bold finding in our survey is related to the approximation of donors in the two clusters of our research. A rather significant percentage, 10.5%, summarizes the current organ donor status for students, while a rather low percentage of 3.5% is the relevant rate for the general population. The constructive comment is that there are many more things to be developed in order to maintain a sustainable Organ Donor Registry status in Saudi Arabia.

Our research also focused on the collection of various data for the analysis of organ donation in Saudi Arabia. In the next section, we provide several tabular overviews for more detailed results in our research.

### Detailed Overview of Results

#### Demographics of the Study

In this section, we summarize the key demographics for our survey.

#### General Attitude Toward Organ Donation

We provide a summary of responses related to the general inquiry (Table 2) about organ donation as it was clarified in our research.

#### Knowledge About Kidney Donation (Detailed Data Overview)

In this section, Table 3 summarizes the knowledge and perceptions of our respondents about kidney donation.

#### Attitude Toward Kidney Donation (Detailed Data Overview)

In an effort to provide a detailed data overview of our research regarding the attitude of the respondents toward kidney donation, we provide in Table 4 below the key aspects of the phenomenon as was confirmed in our research study.

**Table 4:** Attitude toward kidney donation.

**Attitude**	**Agree *N* (%)**	**Neutral *N* (%)**	**Disagree *N* (%)**	***P*-value**
1. Kidney donation is a good thing and should be promoted				0.197
Students	258 (82.4)	53 (16.9)	2 (0.6)	
General population	290 (78)	81 (21.8)	1 (0.3)	
2. Registering as kidney donor could save somebody's life				0.371
Students	292 (93.3)	18 (5.8)	3 (1)	
General population	342 (91.9)	23 (6.2)	7 (1.9)	
3. Saudi as well as Non-Saudi residents should be automatically included on the Organ Donor register of Saudi, with the ability to refuse if they wish				0.092
Students	178 (56.9)	80 (25.6)	55 (17.6)	
General population	232 (62.4)	90 (24.2)	50 (13.4)	
**I would be more willing to register as a kidney donor:**				
4. If I knew that my family would have no objection to allowing donation of my kidney at the time of my death				0.092
Students	214 (68.4)	71 (22.7)	28 (8.9)	
General population	237 (63.7)	86 (23.1)	49 (13.2)	
5. If I knew more about what kidney transplant is and how it is done				0.699
Students	208 (66.5)	83 (26.5)	22 (7)	
General population	249 (66.9)	88 (23.7)	35 (9.4)	
6. If more information was available about the viewpoint of my religion with regard to				0.963
Students	211 (67.4)	81 (25.9)	21 (6.7)	
General population	253 (68)	91 (24.5)	28 (7.5)	
7. If I knew where I could register				0.780
Students	198 (63.3)	83 (26.5)	32 (10.2)	
General population	225 (60.5)	114 (30.6)	33 (8.9)	

#### Beliefs in Relation to Kidney Donation (Detailed Data Overview)

We also focused our research on the understanding of beliefs of Saudi residents and non-Saudi residents regarding kidney donation. Table 5 below summarizes the key aspects and findings. We also plan to run another research and survey soon on cultural and religious issues associated with organ donation.

**Table 5:** Beliefs in relation to kidney donation.

**Beliefs**	**Agree *N* (%)**	**Neutral *N* (%)**	**Disagree *N* (%)**	***P*-value**
1. I think my donation whether living or after death is going to impact my life after death in a good way				0.412
Students	244 (78)	46 (14.7)	23 (7.3)	
General population	296 (79.6)	56 (15.1)	20 (5.4)	
2. Kidney donation is an act which will be rewarded by God				0.043
Students	267 (85.3)	38 (12.1)	8 (2.6)	
General population	337 (90.6)	29 (7.8)	6 (1.6)	
3. Kidney retrieval process after death may cause body disfigurement				0.001
Students	92 (29.4)	127 (40.6)	94 (30)	
General population	59 (15.9)	143 (38.4)	170 (45.7)	
4. You do not find many opportunities to register as a kidney donor in Saudi Arabia				0.004
Students	144 (46)	118 (37.7)	51 (16.3)	
General population	125 (33.6)	172 (46.2)	75 (20.2)	
5. Kidney donor registration is a time-consuming process				0.015
Students	98 (31.3)	155 (49.5)	60 (19.2)	
General population	134 (36)	196 (52.7)	42 (11.3)	
6. While registering for kidney donation, you may not get answers for all your questions				0.039
Students	107 (34.2)	149 (47.6)	57 (18.2)	
General population	155 (41.7)	163 (43.8)	54 (14.5)	
7. You are not healthy to donate				0.030
Students	90 (28.8)	87 (27.8)	136 (43.5)	
General population	115 (30.9)	138 (37.1)	119 (32)	
8. Your age is not fit for donating your kidney				0.001
Students	54 (17.3)	88 (28.1)	171 (54.6)	
General population	89 (23.9)	135 (36.3)	148 (39.8)	
9. Operation procedure for procuring kidney is discouraging				0.575
Students	106 (33.9)	156 (49.8)	51 (16.3)	
General population	130 (34.9)	166 (44.6)	76 (20.4)	
10. You are worried that kidney donation might leave you weak and disabled				0.001
Students	183 (58.5)	54 (17.3)	76 (24.3)	
General population	112 (30.1)	98 (26.3)	162 (43.5)	
11. Emotions of your family members while kidney is being taken make you feel concerned				0.484
Students	175 (55.9)	61 (19.5)	77 (24.6)	
General population	185 (49.7)	102 (27.4)	85 (22.8)	

#### Correlation of Key Aspects (Detailed Data Overview)

In Tables 6–8 below, we provide key facts for the correlation and the value perception of organ donation in terms of willingness and barriers. This will also be a direction for a future research that will exploit the key findings of the current survey.

**Table 6:** Correlation between knowledge and willingness.

**Knowledge about kidney donation**	**Yes *N* (%)**	**No *N* (%)**	***P*-value**
**Are you willing to donate your kidney?**			
Have you ever attended organ donation promotion campaigns in Saudi Arabia?			
Students	34 (53.1)	30 (46.9)	0.001
General population	13 (72.2)	5 (27.8)	0.001
What does organ/tissue/blood donation mean to you?			
Students	68 (28.8)	168 (71.2)	0.022
General population	53 (20.7)	203 (79.3)	0.852
Does your religion allow organ donation?			
Students	86 (36.8)	148 (63.2)	0.003
General population	65 (24.9)	196 (75.1)	0.002
Do you know anyone who has donated a kidney?			
Students	30 (40.5)	44 (59.5)	0.082
General population	36 (24)	114 (76)	0.238
Do you know that donating a kidney is safe?			
Students	69 (37.5)	115 (62.5)	0.018
General population	56 (30.3)	129 (69.7)	0.001
Prohibits any buying or selling of organs			
Students	84 (33.6)	166 (66.4)	0.316
General population	62 (25.3)	183 (74.7)	0.004
Provides access to transplant facility for all nationalities equally			
Students	58 (35.2)	107 (64.8)	0.250
General population	56 (25.3)	165 (74.7)	0.012
Gives donated organs from deceased donors to the first person on the waiting list regardless of nationality			
Students	67 (33.2)	135 (66.8)	0.646
General population	62 (25.3)	183 (74.7)	0.004

**Table 7:** Correlation between attitude and willingness.

**Attitude**	**Yes *N* (%)**	**No *N* (%)**	***P-*value**
**Are you willing to donate your kidney?**			
Kidney donation is a good thing and should be promoted			
Students	92 (35.7)	166 (64.3)	0.005
General population	69 (23.8)	221 (76.2)	0.011
Registering as kidney donor could save somebody's life			
Students	100 (34.2)	192 (65.8)	0.007
General population	77 (22.5)	265 (77.5)	0.040
Saudi as well as Non-Saudi residents should be automatically included on the Organ Donor register of Saudi, with the ability to refuse if they wish			
Students	69 (38.8)	109 (61.2)	0.025
General population	64 (27.6)	168 (72.6)	0.001

**Table 8:** Correlation between beliefs and willingness.

**Beliefs**	**Agree *N* (%)**	**Neutral *N* (%)**	**Disagree *N* (%)**	***P*-value**
I think my donation whether living or after death is going to impact my life after death in a good way				0.412
Students	244 (78)	46 (14.7)	23 (7.3)	
General population	296 (79.6)	56 (15.1)	20 (5.4)	
Kidney retrieval process after death may cause body disfigurement				0.001
Students	92 (29.4)	127 (40.6)	94 (30)	
General population	59 (15.9)	143 (38.4)	170 (45.7)	
You do not find many opportunities to register as a kidney donor in Saudi Arabia				0.004
Students	144 (46)	118 (37.7)	51 (16.3)	
General population	125 (33.6)	172 (46.2)	75 (20.2)	
Kidney donor registration is a time-consuming process				0.015
Students	98 (31.3)	155 (49.5)	60 (19.2)	
General population	134 (36)	196 (52.7)	42 (11.3)	
You are not healthy to donate				0.030
Students	90 (28.8)	87 (27.8)	136 (43.5)	
General population	115 (30.9)	138 (37.1)	119 (32)	
You are worried that kidney donation might leave you weak and disabled				0.001
Students	183 (58.5)	54 (17.3)	76 (24.3)	
General population	112 (30.1)	98 (26.3)	162 (43.5)	
Emotions of your family members while kidney are being taken make you feel concerned				0.484
Students	175 (55.9)	61 (19.5)	77 (24.6)	
General population	185 (49.7)	102 (27.4)	85 (22.8)	

In the next section, we try to integrate and to synthesize the key findings of our research.

## Discussion

The historical journey of renal transplantation in Saudi Arabia was started with the first successful kidney transplantation from a live donor in 1979 that took place in Riyadh Military Hospital. Because religion plays a significant role in the laws and legislations of Saudi Arabia, Islamic religious opinion has to be approved with the concept of brain death and organ donation from both living and cadaveric donors before organ transplantation is authorized. The Islamic approval in Saudi Arabia came in 1982, where the Council of Senior Scholars issued a decision that permits organ donation in both live and dead donors, and this announcement was followed by the decision of Islamic Jurisprudence about the definition and permission to switch off the ventilator from brain dead individuals. Afterwards, the Fiqh Academy of Muslims World League, Mecca, Saudi Arabia, sanctioned in the eighth session organ donation and transplantation as being compatible with Islam in 1985 (24–26). After that, renal transplantation service in Saudi Arabia carried out several developmental phases to establish the National Kidney Foundation in the same year (27). In December 14, 1992, one of the distinctive moves has taken place regarding organ donation in Saudi Arabia where the National Kidney Foundation was upgraded and got renamed as the Saudi Center for Organ Transplantation (SCOT), which is now considered a multiorgan donation center that supervises all national transplant activities in Saudi Arabia. Nowadays, there are 13 transplantation centers in Saudi Arabia with additional centers providing follow-up post-transplant (28).

Although many of our population, when asked about their willingness to register as kidney donors in Saudi Arabia, answered that they are not willing to donate their kidney, it appears that students showed higher desire to donate their kidney (32.2%) when compared with the general population who showed lower desire (21%). On the other hand, a similar study that was done in Turkey showed a willingness rate of 58.7% among medical students, which is significantly higher than the willingness rate among health science students at King Saud bin Abdulaziz University (29). The total willingness rate among the sample study is 26.1% in comparison to 66%, which represents the willingness rate among the Nigerian population (30).

Several factors play a big role affecting the willingness of these students. In a research that was done in Nigeria, their results reveal that high level of knowledge in regard to transplantation was positively influential on the willingness to donate a kidney among the public (2). Similarly, in our study, a major factor that showed increase in the likelihood of these students to donate their kidney is their knowledge about kidney donation. Also, the mean score of knowledge was higher in students when compared with the general population. Medical students seemed more knowledgeable about the concept of organ/tissue transplant and meaning of death than the general population. This was the opposite when it came to a study done by Edwards et al. in which only 28% of their students were able to identify the concept of brain death correctly, which was significantly lower than that of the general population (34%). Moreover, in the same study, the readiness to donate among medical students was 32% (4). The rate of willingness of medical students to donate in another study that was done in India was significant (89%); however, those students were not aware of any organizations for organ donation, and only 25% of them were able to understand the concept of brain death correctly (7). On the contrary, a study was done in Pakistan that illustrated that level of knowledge did not correlate significantly and positively with the will to donate among medical students (6).

A further interesting point to discuss regarding the knowledge is that students revealed a low rate of correctly knowing the current regulations and laws in Saudi Arabia regarding organ donation, which indicates lack of knowledge about legal facts in regard to this topic when compared with the general population who were more well-informed about the matter. This was similar to a study that was done in China that reported that medical students' poor rate of properly identifying rulings concerning organ donation reflect them being unlettered when it comes to legal facts about it (3).

Moreover, in spite of the fact that more of the general population stated that they knew someone in their life who had donated a kidney before (40.3%) than students (23.6%), students showed a significant relationship between knowing someone who have donated before with higher willingness than the general public. The association between willingness and the knowledge of allowance of organ donation in religion and the safety of organ donation was similar between the two targeted groups. Almost 30% of our cohort was not aware that organ donation was approved by Purport of the Senior Ulama Commission, similar to the finding from Southwest Nigeria, whereby religious beliefs were found to be influential on the willingness of organ donation (2).

While the majority of our study population answered that they think that Islam allows organ donation, still many people in the Muslim community are ambivalent about it.

There is still lack of unanimity between people in Muslim communities regarding compatibility of organ donation with Islam despite the plentiful rulings that are in support of it, so it is essential to explore the arguments that are often made both in favor and against organ donation.

Some arguments that are made against this topic are often developed due to Muslims being confused or having some questions regarding some aspects of organ donation that are not answered clearly. The first argument speaks against the principle of “Necessity Overrules Prohibition,” saying that not all organ transplantations are considered an absolute necessity due to the presence of other available treatment options. Muslims who agree with this statement often give an example of kidney transplantation not being the only treatment option for ESRD with the existence of other modalities including dialysis. Another conflict pointed out by these groups of Muslims is that organ transplantation interferes with the will and predetermination of God (Qadar). Moreover, many Muslims also believe that every person should present on the Day of Judgment with their organs intact since these organs will testify before God about the person's deed during their life. An example of a popular figure who spoke against organ donation, specifically kidney donation, in Islam is Muhammad Metwali Al Shaarawy, saying “How can you give a kidney that you yourself do not own?” (24, 31).

A key argument in favor of organ donation, as mentioned previously, is the principle of “Necessity Overrules Prohibition.” Basing their argument on it, those groups of Muslims claim that donating an organ in life or death for the good deed of saving a life is the priority. The following is the frequently quoted line from the Qur'an that they believe support this claim: “If anyone saved a life, it would be as if he saved the life of the whole people” Surah Al Ma'idah-5:32. Furthermore, a saying of Prophet Muhammad “Make use of medical treatment, for Allah has not made a disease without appointing a remedy for it” is often used in support of organ donation (24, 31).

Attending organ donation campaigns is a common factor between the students and general population, which positively influences the willingness to donate a kidney. Beside the knowledge, it is the only factor that had a great association with being willing to donate and taking a step forward by registering at SCOT as a kidney donor among students. The considerable effect of campaigns on raising awareness and knowledge nourishment about kidney donation in our society is evident by the significant correlation between attending campaigns and being more willing to hold an organ donor card that was found in this study. In fact, the impact of organ promotion campaigns was observable in our community through the progressive improvement that has been accomplished in the past 10 years. According to the annual reports that were published by SCOT, one of the strategies that were used to increase organ donation pool is expanding educational activities with a primary goal to clarify the concept of organ donation and brain death in Saudi community by expert supervised campaigns and maximizing media involvement. Relating these efforts to the study findings, two possible causes of the limited improvement in donation in Saudi Arabia were identified.

The first issue is that the campaigns that were done in Saudi Arabia did not illustrate and appropriately clarify the safety of donations and the Islamic point of view, which were found to be significant in association with the willingness as discussed previously. Similarly, in a study that was done in Nigeria, it highlights the importance of discussing donation safety issues (2). Another study conducted in Pakistan revealed the significance of clarifying the religious and legal ambiguities through public campaigns (6). The second issue, according to SCOT annual reports, is that most of the campaigns and awareness-raising activities were restricted to hospital corridors, and this could be a contributing factor to the high knowledge mean score among the health science students.

The same as knowledge, a positive attitude toward kidney donation greatly correlates with kidney donation willingness among both populations. Surprisingly, there was no difference in the attitude mean score between them. Nevertheless, in a similar study that was done in Germany, they concluded that the high organ donation willingness rate among medical students was explained by the positive impact of medical education on both attitude toward and knowledge about organ donation (8). Unexpectedly, 56.9 and 62.4% of the students and the general population, respectively, agreed on automatic registry in SCOT, with the ability to refuse if they wish. The high rate of agreement on automating the organ registry system could be explained by not having to face organ donation barriers that was shown in this study such as family disagreement and carrying the burden of religious disapproval. This is a promising finding to consider because it simply means that overcoming these barriers by raising awareness and knowledge improvement could be a turning point in the future of the organ donation system in Saudi Arabia. The study that was done by Edwards et al. stated that family disapproval was the main barrier of organ donation among the medical students (4). Moreover, in a study that was done in India, post-graduate medical students declared that too many hassles with regard to organ donation procedures was the main reason for being unwilling to hold an organ donor card (7).

Despite the fact that students are more willing to donate their kidney, the general population scored higher level of positive beliefs toward kidney donation. The general population's willingness toward kidney donation was positively influenced by their belief that SCOT offers plenty of registration opportunities that are not time-consuming and that their family's emotions are not considered an obstacle, compared with the student population who believe that kidney donation is going to impact their life after death in a good way and that the kidney procurement process will not leave them weak or disabled.

According to annual reports that were published on SCOT's official website, there is a progressive improvement in organ donation over the past decade in Saudi Arabia. In the annual report of 2015, they have stated that there was an increase in the number of organ donation as 762 organs were transplanted successfully over the year. Several methods used to reactivate the kidney donation program were discussed throughout the report. One of the important aspects is to increase public knowledge and awareness by, for example, clarifying the concept of brain death and donation through brochures, awareness-raising lectures and activities, and campaigns. We recommend continuing SCOT efforts to optimize the local transplantation program with more focus on delivering them to different sectors of society. Furthermore, Internet was the most popular method through which the study sample populations have heard about organ donation from, followed by social events and television among the students and the same but in a reversed order among general participants. This emphasizes the importance of involving the media in clarifying kidney donation-related misconceptions; promoting after-death organ donation; introducing SCOT, registry process, and form to society; and clearing up Saudi organ donation policies. We suggest to make use of the national day for organ donation, which is on August 13 in Saudi Arabia, by enhancing the knowledge of organ donation policy in Saudi Arabia and the means of registration, that is, through social media, advertisements, videos, and campaigns. We also recommend clarifying the religious aspect toward donation acceptance among the general population by benefiting from religious gatherings and Friday speech. One of the valuable strategies is to work on minimizing the difficulties experienced during the organ donation process, including delay in the funerals and time spent on the donation registry process. Another suggestion is to fill up an organ donation card simultaneously with obtaining a driver's license to increase the number of organ donors or to at least increase the accessibility and awareness of organ registry. We emphasize on evolving the application of the Kidney Exchange Program with the collaboration of gulf countries. In 2010, the Australian and New Zealand Paired Kidney Exchange (ANZKX) Program was utilized, aiming to increase the access to life-saving kidney transplants. According to the Australian Ministry of Health, the collaboration with New Zealand enables further growth and success of the organ donation program and more transplant opportunities for Australians and New Zealanders. The cornerstone principle of this method is to increase the chance of finding matches for people who have a relative or friend who is willing to donate, but unable to do so because their blood or tissue type is not compatible. It would be promising to improve such a program in an expandable manner over several countries; however, to be realistic, several considerations need to be addressed. For example, to establish the necessary transport arrangements, multiple government agencies and airline companies must implicate an effective and professional cooperation.

One of the limitations of our study is the discrepancy between the number of females and males, and that could be attributed to the increased numbers of females present in the targeted malls the for general population, while for the student population, female students were more cooperative to engage in our study. In addition, the College of Nursing at KSAU-HS only accepts female students, which also contributed to the increased number of females in this study. Another limitation is selection bias, where the participants decided to participate because they were interested in the subject of kidney donation.

## Conclusion

The total willingness rate to donate kidney among health science students at KSAU was higher than the general population. However, when student's willingness rate was compared to other health science students in international literature, it was relatively low. More importantly, the total number of organ donation registry in the total sample population was significantly low (6.3%). This study illustrates that King Saud bin Abdulaziz University students are more knowledgeable compared to the general population, which positively correlates with kidney donation willingness and being registered as a kidney donor. Moreover, more positive attitudes and beliefs toward kidney donation were associated with higher willingness toward organ donation among both health science students and the general population. Effective measures should be taken to educate and increase the knowledge among both populations. The concept of brain death and a religious point of view in regard to organ donation are important points to be clarified.

## Data Availability Statement

The raw data supporting the conclusions of this article will be made available by the authors, without undue reservation.

## Ethics Statement

The studies involving human participants were reviewed and approved by King Saud bin Abdulaziz University. The patients/participants provided their written informed consent to participate in this study.

## Author Contributions

All authors listed have made a substantial, direct and intellectual contribution to the work, and approved it for publication.

## Conflict of Interest

The authors declare that the research was conducted in the absence of any commercial or financial relationships that could be construed as a potential conflict of interest.
